# The Solvent Process of Glycerin

**Published:** 1858

**Authors:** John S. Blockey


### The Solvent Powers of Glycerin.

BY JOHN S. BLOCKEY.

Some time back, having occasion to experiment with the disulphate of quinine, I accidentally discovered that glycerin, if gently heated, will dissolve more than 8 grains per fluid drachm, or about one-twelfth of its weight of this salt; this fact will render glycerin a valuable vehicle for the therapeutical administration of quinine, as I noticed in a communication to the "Lancet," a few weeks ago. I have since found that glycerin appears to possess properties that may, perhaps, give it a place among the usual disolving substances; for instance, salicin soluble in 22 parts by weight of cold water, and in 30 parts of 80 per cent. alcohol, will disolve in 8 parts of cold glycerin; santonin, soluble in 250 parts of boiling water, and in 3 of boiling alcohol, will dissolve in 18 parts of boiling glycerin; the solution however, becomes thick and almost solid, with only one grain of santonin in 36 of glycerin on cooling, and a saturated boiling solution of santonin in glycerin, when cold may be inverted without loss.

Strychnia is soluble in 80 parts of boiling glycerin, but very slightly in cold.

From these experiments it appears that many substances are soluble in glycerin, in a very different ratio to their solubility in water, etc. Iodide of lead dissolves sufficiently in boiling glycerin, to cause the solution to become turbid on cooling. Aconitina is scarcly soluble at all in this medium. May not glycerin be found a solvent for many other comparatively insoluble substances, both in the inorganic and in the organic kingdoms?

I invariably heat the glycerin to give it greater fluidity, and the quantity that may be thus dissolved, the solution remaining clear on cooling, I estimate as the quantity soluble in cold glycerin.—*London Chemist.*

